# Evolution of insecticide resistance and its mechanisms in *Anopheles stephensi* in the WHO Eastern Mediterranean Region

**DOI:** 10.1186/s12936-020-03335-0

**Published:** 2020-07-17

**Authors:** Ahmadali Enayati, Ahmad Ali Hanafi-Bojd, Mohammad Mehdi Sedaghat, Morteza Zaim, Janet Hemingway

**Affiliations:** 1 Department of Medical Entomology and Vector Control, School of Public Health and Health Sciences Research Center, Mazandaran University of Medical Sciences, Sari, Iran; 2 Department of Medical Entomology and Vector Control, School of Public Health, Tehran University of Medical Sciences, Tehran, Iran; 3 Liverpool School of Tropical Medicine, Liverpool, UK

**Keywords:** *Anopheles stephensi*, Resistance, Mechanisms, Iran, Eastern Mediterranean Region

## Abstract

**Background:**

While Iran is on the path to eliminating malaria, the disease with 4.9 million estimated cases and 9300 estimated deaths in 2018 remains a serious health problem in the World Health Organization (WHO) Eastern Mediterranean Region. *Anopheles stephensi* is the main malaria vector in Iran and its range extends from Iraq to western China. Recently, the vector invaded new territories in Sri Lanka and countries in the Horn of Africa. Insecticide resistance in *An. stephensi* is a potential issue in controlling the spread of this vector.

**Methods:**

Data were collated from national and international databases, including PubMed, Google Scholar, Scopus, ScienceDirect, SID, and IranMedex using appropriate search terms.

**Results:**

Indoor residual spaying (IRS) with DDT was piloted in Iran in 1945 and subsequently used in the malaria eradication programme. Resistance to DDT in *An. stephensi* was detected in Iran, Iraq, Pakistan, and Saudi Arabia in the late 1960s. Malathion was used for malaria control in Iran in 1967, then propoxur in 1978, followed by pirimiphos-methyl from 1992 to 1994. The pyrethroid insecticide lambda-cyhalothrin was used from 1994 to 2003 followed by deltamethrin IRS and long-lasting insecticidal nets (LLINs). Some of these insecticides with the same sequence were used in other malaria-endemic countries of the region. Pyrethroid resistance was detected in *An. stephensi* in Afghanistan in 2010, in 2011 in India and in 2012 in Iran. The newly invaded population of *An. stephensi* in Ethiopia was resistant to insecticides of all four major insecticide classes. Different mechanisms of insecticide resistance, including metabolic and insecticide target site insensitivity, have been developed in *An. stephensi.* Resistance to DDT was initially glutathione S-transferase based. Target site knockdown resistance was later selected by pyrethroids. Esterases and altered acetylcholinesterase are the underlying cause of organophosphate resistance and cytochrome p450s were involved in pyrethroid metabolic resistance.

**Conclusions:**

*Anopheles stephensi* is a major malaria vector in Iran and many countries in the region and beyond. The species is leading in terms of development of insecticide resistance as well as developing a variety of resistance mechanisms. Knowledge of the evolution of insecticide resistance and their underlying mechanisms, in particular, are important to Iran, considering the final steps the country is taking towards malaria elimination, but also to other countries in the region for their battle against malaria. This systematic review may also be of value to countries and territories newly invaded by this species, especially in the Horn of Africa, where the malaria situation is already dire.

## Background

Iran is on track to achieve malaria elimination, with zero indigenous cases in 2017 and 2018 [1]. National strategic planning is in place to eliminate the disease. In contrast, in the early 20^th^ Century, there were five million cases of malaria in a country of 18 million people [2, 3]. Now 1% of the 81-million population live in malaria-endemic areas [1] compared with 75% in 1925 [3].

DDT was first used in Iran for malaria vector control in 1945 [2–4]. It was the insecticide of choice during the national malaria eradication campaign starting in 1956 following the Global Malaria Eradication Programme (GMEP) guidelines [2–7]. Insecticide resistance prompted a series of insecticide changes over time, from DDT to dieldrin, malathion, propoxur, pirimiphos-methyl, lambda-cyhalothrin, and deltamethrin to the present time [3, 8–20].

Iran was not alone in the battle against malaria. Almost all malaria-endemic countries outside sub-Saharan Africa embraced the GMEP using the main intervention of indoor residual spraying (IRS) [6, 7]. Resistance to several insecticides of all major classes has been reported in *Anopheles stephensi* in other countries of the WHO Eastern Mediterranean Region (EMR), including Saudi Arabia [21, 22], Iraq [23, 24], Afghanistan [25–28], and Pakistan [29, 30]. Resistance to DDT, dieldrin, malathion, and recently pyrethroid insecticides, was reported from different states of India [31–41].

Involvement of several enzyme groups in insecticide resistance is evident in many insects, including mosquitoes [42–46]. Several mechanisms including metabolic and insecticide target site insensitivity are involved in insecticide resistance in *An. stephensi* from different countries, each of which has its own operational significance in vector control [26–28, 31, 33–36, 38, 39, 42, 47, 48].

Recently, *An. stephensi* expanded its distribution range into Sri Lanka and the Horn of Africa, where it has been detected in Djibouti, Ethiopia and Sudan [49–54]. The species in Ethiopia was highly resistant to DDT, malathion, pirimiphos-methyl, bendiocarb, propoxur, deltamethrin, and permethrin [54]. Resistance to insecticides was also detected in *An. stephensi* in Sri Lanka [55]. This expansion of the distribution range in Asia and especially in Africa is a cause for concern so that the WHO organized a technical consultation meeting to assess the situation in 2019 [52]. This evidence-based analysis of the literature on insecticide resistance in *An. stephensi* in Iran and the countries of the EMR, especially the countries and territories recently invaded by *An. stephensi*, is designed to inform national malaria programmes for appropriate deployment of vector control interventions and timely management of insecticide resistance in line with the WHO Global Plan for Insecticide Resistance Management (GPIRM) [56].

## Methods

This is a systematic review of all literature and evidence available for insecticide resistance in *An. stephensi.*

### Inclusion criteria

All studies on insecticide resistance performed on adults and larvae of *An. stephensi* from WHO EMR countries using WHO standard kits and procedures for mosquito susceptibility tests were included regardless of dates and language. Studies using filter papers impregnated with only WHO-recommended discriminating concentrations of insecticides for adult mosquitoes or WHO-discriminating concentration for larvae detailed in Table 1, were included in the review. Studies using laboratory-selected strains were included only where they reported the underlying resistance mechanisms. India and Ethiopia are not WHO member states of the EMR but studies from the former are included as there are several reports in the literature on the resistance of the species to different insecticides and its underlying mechanisms, and the latter is included as the insecticides susceptibility status in *An. stephensi* in countries newly invaded by the species is important.

**Table 1:** Susceptibility status (*R* resistant, *RC* resistance to be confirmed, *S* susceptible, *NR* no report) of *Anopheles stephensi* to selected insecticides in Iran (2020)

Province	Organochlorines		Organophosphates		Carbamates		Pyrethroids	
	DDT 4%	Dieldrin 4%	Malathion 5%	Temephos 0.25 mg/l	Propoxur 0.1%	Bendiocarb 0.1%	Lambdacyhalothrin 0.05%	Deltamethrin 0.05%
Fars^a^	R	R	R	NR	S	NR	NR	NR
Hormozgan	R^b^	R	S	S	RC	R	R	RC
Kerman	RC	NR	S	S	S	R	S	S
Sistan and Baluchestan	R	S	RC	NR	R	RC	R	R

^a^As malaria has long been eliminated from this province, the susceptibility bioassays are rather old

^b^There is only one outlier study to this generalization which needs confirmation

### Exclusion criteria

Studies and experiments using insecticides and concentrations or test kits other than those approved by WHO, including those using plant extracts, dose–response bioassays, bioassays performed on nets and residual bioassays, were excluded from this study.

### Search strategy, study selection, data extraction, and synthesis

The search period was from 1 January, 1925 to 10 March, 2020. The following databases were searched for relevant studies using appropriate search terms and strategy: PubMed, Google Scholar, Scopus, ScienceDirect, SID, and IranMedex. Relevant conference proceedings were checked and the reference lists of all included studies identified by the above methods were also hand searched.

The search results were primarily screened based on the title and abstract followed by a second round of screening through the full text to select relevant studies for inclusion in the review. Blank tables were designed in Microsoft Excel and used to extract the relevant data from the included studies. The extracted data were used to build a chronological history of insecticide resistance and its underlying mechanisms in *An. stephensi.* The insecticide resistance data were used to plot separate maps indicating the distribution of resistance to insecticides. WHO criteria and classification for insecticide resistance [57] were considered for mapping. All maps were prepared in ArcGIS 10.5 at the district level for Iran and province/state level for the region. Shape files of Iran were provided by the National Cartography Centre, while the shape files for the region were downloaded from the Natural Earth website (www.naturalearthdata.com).

## Results

**Fig. 1:**
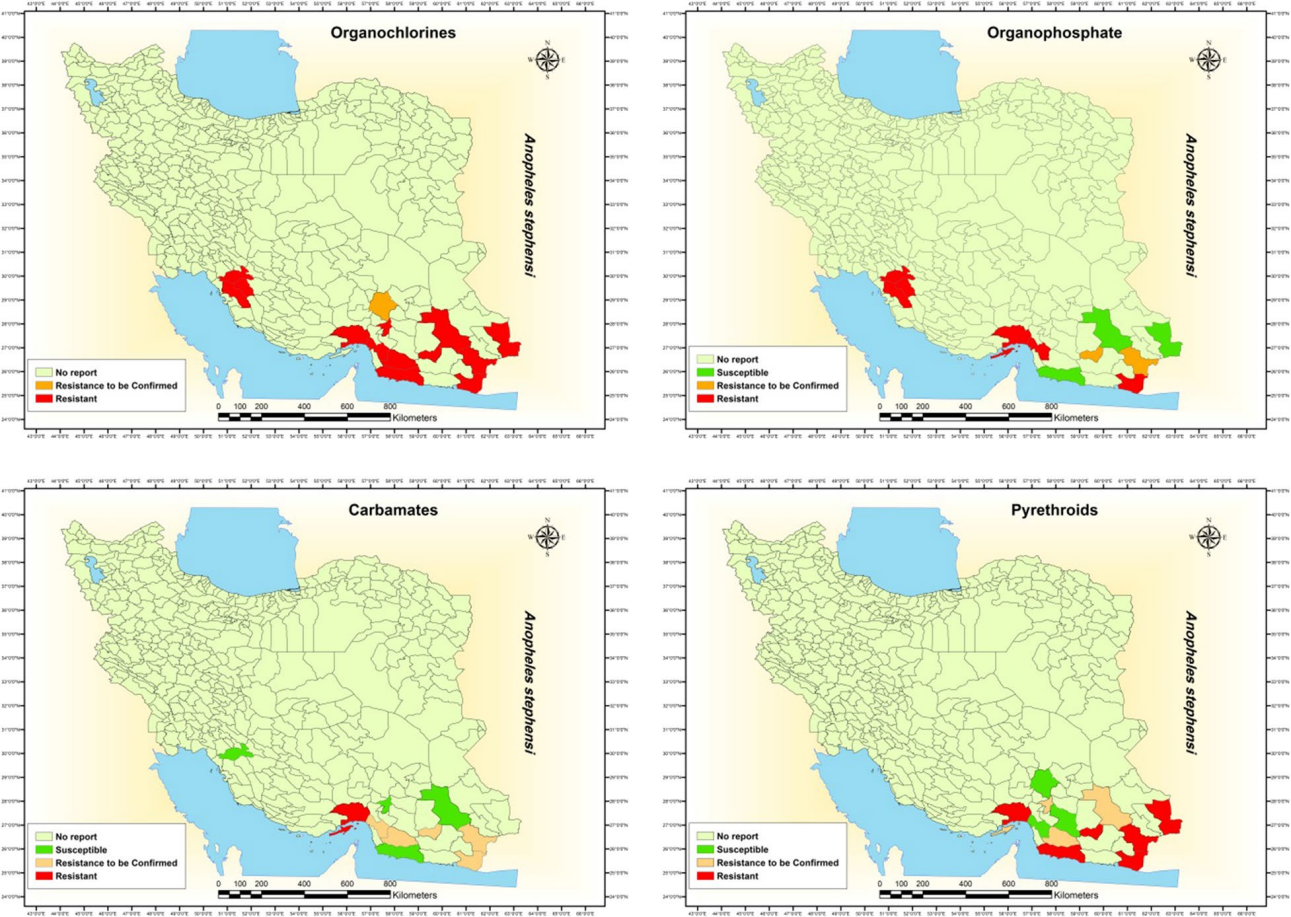
Insecticide resistance status of *An. stephensi* to different insecticides at district level in Iran

After its first application as indoor residual spraying (IRS) for malaria control in Iran in 1945 [2, 3, 6, 7], DDT was used until 1957 when *An. stephensi* became resistant to it [8] followed by dieldrin use and development of resistance in 1959–1960 [9–12]. In later years susceptibility to DDT and dieldrin decreased further in the 1970s [11, 15, 58]. Susceptibility to these insecticides gradually rose to the final years of the Millennium [19, 20, 59, 60]. Over the years, DDT resistance persisted but that of dieldrin decreased more rapidly changing its susceptibility status to tolerance [61], and later to complete susceptibility [62–64]. However, from 2010, susceptibility of *An. stephensi* to DDT and dieldrin decreased, may be due to the emergence of resistance to pyrethroid insecticides [65–71]. The susceptibility status of *An. stephensi* to organochlorine insecticides in different provinces of Iran is summarized in Table 1 and mapped in Fig. 1.

Due to the double resistance of *An. stephensi* to DDT and dieldrin, malathion IRS was used in malaria vector control in Iran since 1968 [14]. Susceptibility bioassays in 1975 showed that the species was susceptible to malathion with a mortality of 99% [11, 15]. However, the species developed resistance to malathion in 1976 [17] and remained so for some years [58]. Regardless of a couple of instances of mortality of less than 95% in 2011 [64] and 90% in 2015 [72], the species proved to be susceptible to malathion-discriminating concentration since 1982 in its entire range in southern Iran [19, 59–62, 65, 66, 68, 69, 71]. Interestingly, resistance to malathion in adult *An. stephensi* did not decrease the susceptibility of its larvae to temephos within the same organophosphate (OPs) class [73]. Several susceptibility bioassays on *An. stephensi* larvae to temephos showed complete susceptibility [60, 61, 68, 74, 75]. The susceptibility status of *An. stephensi* to OPs in different provinces of Iran is summarized in Table 1 and mapped in Fig. 1.

Since 1978, propoxur was used as IRS for malaria vector control in Iran [18, 19]. Propoxur bioassays revealed complete susceptibility in later years [19, 58]. Although subsequently the susceptibility status to propoxur and bendiocarb changed to tolerance [20, 72, 75], susceptibility to propoxur is restored while resistance to bendiocarb developed in the species in recent years [59–61, 64, 68, 69, 76]. The susceptibility status of *An. stephensi* to carbamate insecticides in different provinces of Iran is summarized in Table 1 and mapped in Fig. 1.

Pirimiphos-methyl replaced propoxur in 1991 until it was replaced with lambda-cyhalothrin plus propoxur in 1994 [19, 20]. Since 2003, IRS continued using only deltamethrin and lambda-cyhalothrin until 2011. Since 2012, deltamethrin plus bendiocarb were used in an alternative manner for pyrethroid insecticide resistance management [3]. Follow-up bioassays from 2004 to 2010 revealed complete susceptibility to these insecticides in *An. stephensi* from its entire range in southern areas of Iran [59–64, 66]. The first sign of reduced susceptibility to deltamethrin with 97% mortality emerged in 2010 in an area in the south of Iran [77], followed by the development of resistance to lambda-cyhalothrin and resistance to be confirmed to deltamethrin [67, 68]. Despite the results of a couple of studies in 2014 and 2015 showing complete susceptibility of *An. stephensi* to deltamethrin [69, 72], decreased susceptibility to pyrethroid insecticides were observed in later years [70–72, 75, 78]. In included studies, susceptibility of *An. stephensi* to many pyrethroid insecticides were determined, however, only the susceptibility status of *An. stephensi* to deltamethrin and lambda-cyhalothrin in different provinces of Iran is summarized in Table 1 and mapped in Fig. 1.

### Insecticide resistance in *Anopheles stephensi* in other WHO/EMR countries, plus India, Sri Lanka and Ethiopia

In Afghanistan, there was evidence of resistance in *An. stephensi* to DDT, malathion, bendiocarb, deltamethrin, and permethrin in 2011 [25]. Although there are data supporting the susceptibility of the species to bendiocarb and permethrin in some provinces, resistance or resistance to be confirmed has been detected in *An. stephensi* to aforementioned insecticides in most provinces of Afghanistan in the follow-up studies performed in 2014, 2016 and 2017 [26–28, 79]. *Anopheles stephensi* from Lahore, Pakistan was resistant to DDT and malathion [80]. The species proved to be resistant to DDT, malathion, deltamethrin and lambda-cyhalothrin in South Punjab [81]. In another study in Punjab Province, the species was found to be significantly resistant to DDT, dieldrin and malathion while being susceptible to permethrin, deltamethrin and fenitrothion [29]. In a follow-up study in Punjab Province, the species showed resistance to lambda-cyhalothrin, cyfluthrin and cypermethrin [30]. Therefore, *An. stephensi* in Pakistan is resistant to DDT, dieldrin, malathion, and pyrethroid insecticides [79, 82].

Not much is known about the susceptibility status of *An. stephensi* from Iraq. In studies in 1957 and 1980, the species was resistant to DDT, dieldrin and malathion in the country [23, 80]. In India, *An. stephensi* is resistant to DDT in Delhi, Gujarat, Rajasthan, Kerala and Madhya Pradesh whereas it is susceptible to DDT in Karnataka. The species is resistant to malathion in Delhi, West Bengal, Goa, Gujarat, Rajasthan, and Karnataka, susceptible in Kerala and Maharashtra, and resistant to be confirmed in Madhya Pradesh. To deltamethrin, the species is resistant in Karnataka, resistant to be confirmed in Gujarat but susceptible in Kerala, Delhi, Uttar Pradesh, West Bengal and Rajasthan [31–33, 36, 39–41, 79, 82, 83]. In a recent study in Sri Lanka, the species is resistant to DDT, malathion and deltamethrin [55].

**Fig. 2:**
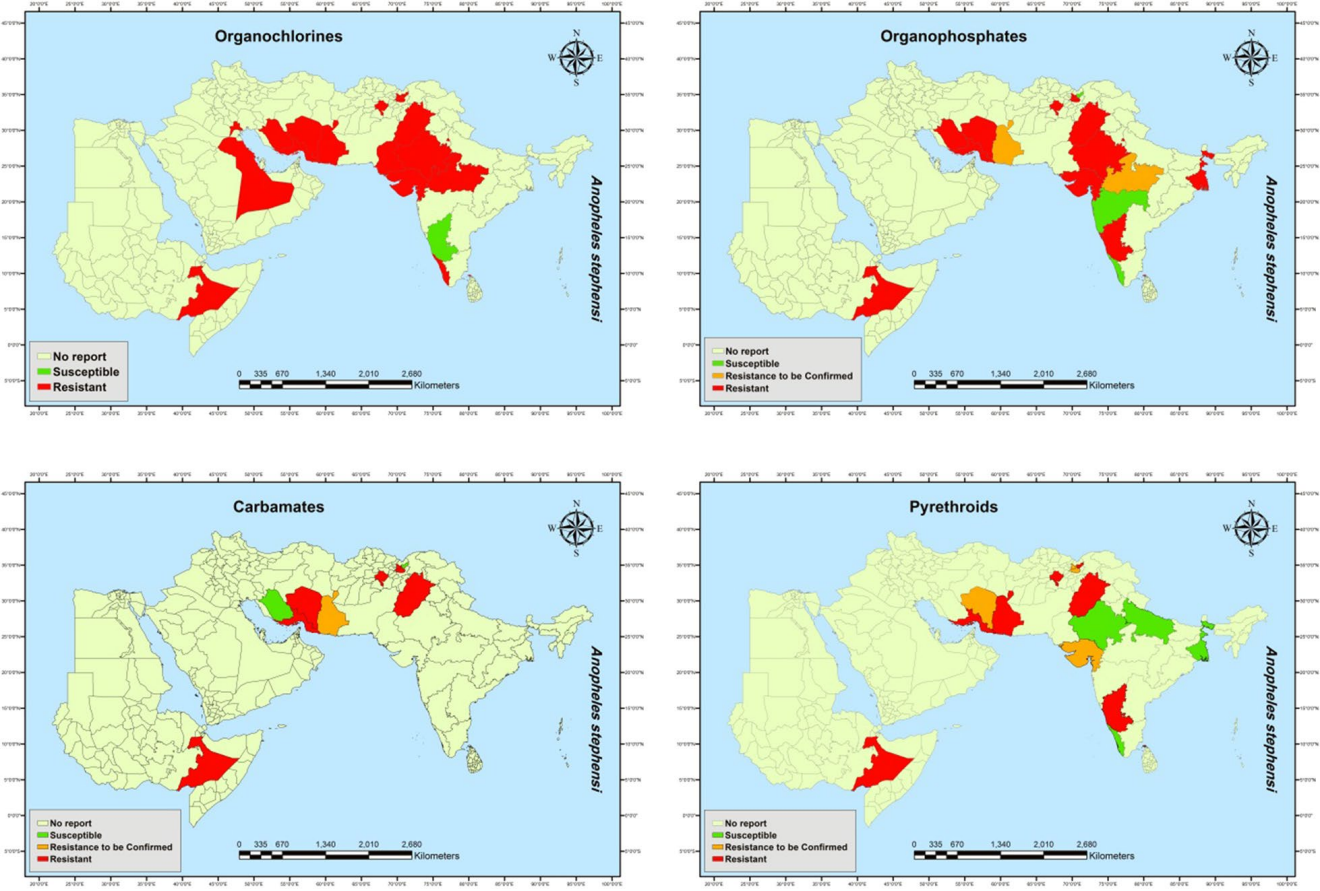
Susceptibility ststus of *An. stephensi* to different insecticide groups in EMR

In Ethiopia, *An. stephensi* was resistant to insecticides of all major classes. Mortality after exposure to the discriminating concentrations of DDT, malathion, pirimiphos-methyl, bendiocarb, propoxur, permethrin and deltamethrin were 32%, 32%, 14%, 23%, 21%, 53% and 67%, respectively, revealing relatively high resistance to all those insecticides [54]. The susceptibility status of *An. stephensi* to different insecticides in different states/provinces of different countries is summarized in Table 2 and mapped in Fig. 2.

**Table 2:** Susceptibility status of *An. stephensi* from different states/province of countries in EMR plus India and Ethiopia

Country	State	OC	OP	C	PY	Reference
Afghanistan	Nangarhar	R	R	R	RC	[26–28]
Afghanistan	Ghazni	R	R	R	R	[26–28]
Afghanistan	Kunar	R	S	S	R	[26–28]
Afghanistan	Laghman	R	R	R	RC	[26–28]
Pakistan	Punjab	R	R	R	R, RC, S	[29, 30, 80, 82]
India	Delhi	R	R	NR	S	[31–33, 36, 39–41, 82, 83]
India	Gujarat	R	R	NR	RC	[31–33, 36, 39–41, 82, 83]
India	Rajasthan	R	R	NR	S	[31–33, 36, 39–41, 82, 83]
India	Kerala	R	S	NR	S	[31–33, 36, 39–41, 82, 83]
India	Madhya Pradesh	R	RC	NR	NR	[31–33, 36, 39–41, 82, 83]
India	Karnataka	S	R	NR	R	[31–33, 36, 39–41, 82, 83]
India	West Bengal	NR	R	NR	S	[31–33, 36, 39–41, 82, 83]
India	Goa	NR	R	NR	NR	[31–33, 36, 39–41, 82, 83]
India	Maharashtra	NR	S	NR	NR	[31–33, 36, 39–41, 82, 83]
India	Uttar Pradesh	NR	NR	NR	S	[31–33, 36, 39–41, 82, 83]
Sri Lanka	Jafna	R	R	NT	R	[55]
Iraq	Basrah	R	NR	NR	NR	[23]
Saudi Arabia	Qatif	R	NR	NR	NR	[21]
Ethiopia	Regional State	R	R	R	R	[54]

*O*: organochlorine, *OP* organophosphates, *C* carbamate, *PY* pyrethroid, *R* resistant, *RC* resistance to be confirmed, *S* susceptible, *NR* no repot

### Mechanisms of insecticide resistance in *Anopheles stephensi*

Synergist bioassays on the DDT and permethrin-resistant laboratory strains of *An. stephensi* from Iran using dichloromethyl benzhydrol (DMC) and piperonyl butoxide (PBO) denoted the involvement of glutathione S-transferases (GSTs) and cytochrome p450s rather than target site insensitivity [84–86]. A genomic study on DDT-resistant strain of *An. stephensi* showed that GSTe2 may be an underlying resistance mechanism in DDT resistance in Iranian *An. stephensi* [62].

The most important mechanisms of resistance to temephos in *An. stephensi* larvae were α-esterases, GSTs and AChE insensitive to propoxur rather than being due to mutations in *ace1* gene [87]. In a follow-up study, it was revealed that the microbiota of the mosquitoes may play a role in their susceptibility status to insecticides as the enzymes involved in resistance of *An. stephensi* to temephos were dramatically reduced in resistant strain when treated with antibiotic [88].

Combination of deltamethrin and PBO applied as IRS resulted in higher mortality in *An. stephensi* adults compared with deltamethrin alone in a semi-field trial in southern Iran. This finding not only reveals the involvement of cytochrome p450s, at least in part, in pyrethroid insecticides resistance, but also shows the benefit of adding PBO to pyrethroid insecticides for malaria vector control to manage insecticide resistance in the field [78]. Involvement of GSTs, esterases and cytochrome p450s in DDT and pyrethroid insecticide resistance was also revealed in a study involving *An. stephensi* adults that underwent selection for DDT and cyfluthrin [89].

In a study on the mechanisms of insecticide resistance in *An. stephensi* in Afghanistan, it was revealed that *kdr* mutations were partially involved in the resistance to pyrethroids as well as DDT, however, the frequency of the *kdr* alleles identified in this study did not explain the whole of the resistance phenotype in *An. stephensi* in eastern Afghanistan, suggestive of involvement of biochemical mechanisms [26]. A follow-up study in Afghanistan revealed that reported resistance to pyrethroid and organophosphorus insecticides, and tolerance to bendiocarb are likely to be caused by a range of metabolic mechanisms, including esterases, cytochrome p450s and GSTs combined with insensitivity in AChE [27]. In another study in eastern Afghanistan, a combination of metabolic mechanisms and increased frequency of *kdr* alleles in the mosquito were blamed for elevated resistance [28]. In Pakistan, esterases are involved in malathion resistance in *An. stephensi* [90, 91]. In India, the involvement of different enzyme groups including esterases, GSTs and cytochrome p450s as well as site insensitivity mechanism of *kdr west* and *east* alleles was revealed [31, 33–36, 38, 39, 47, 48]. The site insensitivity resistance mechanisms in *An. stephensi* from Ethiopia was studied but no *kdr* or *ace1* traits were detected, this may imply the involvement of metabolic mechanisms [54].

## Discussion

Although globally continuous progress is being made in malaria control over the decades, the disease is by far the most important killer in the world [1]. Numerous different strategies and measures have been used to combat malaria, however, insecticide use is pivotal to its vector control and to the interruption of transmission [92]. Continued use of insecticides results in selection for insecticide resistance in vectors of diseases, therefore, continuous monitoring of insecticide resistance and its distribution, mechanisms and management are crucially important for sustainable malaria vector control. The WHO has always urged member states to build adequate capacity and capability for routine monitoring and evaluation of insecticide resistance in malaria vectors through developing plan of action [56, 93–97]. This is not only important for countries that are in the ‘control strategy’ phase, but is of paramount importance for those in the ‘elimination’ and even ‘post-elimination phase’ [97].

The premature development of DDT resistance in Iran in 1957 was probably because of the earlier use of DDT in malaria control in Iran starting in 1945, way before its widespread use during the eradication programme in 1956 [3]. It is interesting to note that in later years even with the cessation of DDT use, it took much longer time for the species to increase its susceptibility to DDT in comparison to the trend of regaining susceptibility to malathion. One reason for that might stem from the fact that DDT resistance is a recessive trait whereas resistance to malathion is more like semi-dominant [80]. Susceptibility to DDT decreased again after 2010 which coincided with the gradual emergence of pyrethroid insecticide resistance and may be due to common metabolic resistance mechanism.

Emergence of pyrethroid insecticide resistance itself in *An. stephensi* in Iran is a cause for concern for malaria control and a potential threat to the malaria elimination programme of the country. Insecticide resistance management strategies are recommended to keep the remaining active ingredients in the arsenal of public health. In recent years *An. stephensi* from Iran developed resistance to bendiocarb, an insecticide used in alternation with pyrethroids to manage insecticide resistance. Resistance to malathion detected in 1976 [11] did not last for long and also its cross resistance spectrum did not include temephos [73]. Serious resistance to propoxur was never detected in *An. stephensi* from Iran and the reason for its replacement with pyrethroid insecticides seems to be more of procurement, cost or pre-emptive insecticide resistance management issues.

The speed of resistance development is dependent on the intrinsic (genetic) and extrinsic (selection pressure) factors. While DDT resistance developed relatively fast in some species, e.g., *An. stephensi*, its development in some other species, e.g., *Anopheles funestus* in South Africa where IRS with DDT started in 1946 was rather slow [98]. In 1996, DDT was replaced with pyrethroid [99], which had to be reversed due to the detection of monooxygenase-based pyrethroid insecticide resistance with no cross-resistance to DDT in *An. funestus* in KwaZulu-Natal Province in 2000 [98].

The development of resistance to different insecticides in *An. stephensi* is usually state- or province-specific within a country as other factors, e.g., differential selection pressure and use of insecticide in agriculture are different. For example *An. stephensi* in Kunar Province in Afghanistan is susceptible to bendiocarb so IRS using this insecticide is prescribed for malaria control in this province. Cytochrome p450, involved in pyrethroid insecticide resistance, is the highest in Laghman and Nangarhar Provinces where PBO-nets can be deployed [28]. The same situation is true in Iran as resistance to certain insecticides is province-specific (Table 1). In South Africa, in part of the malarious areas, DDT IRS is used for malaria vector control whereas in other states, different insecticides are being used [1, 98].

*Anopheles stephensi* has recently invaded new territories and countries in Asia and the Horn of Africa [49–54]. In this invasion, the mosquito takes its insecticide resistance heritage with it as in studies on the insecticide resistance in *An. stephensi* in Ethiopia and Sri Lanka, the species was resistant to major groups of insecticides [54, 55]. Therefore it might be worthwhile to identify the origin of the mosquito should it appear in a new territory.

The WHO urges member states to routinely monitor insecticide resistance, its intensity and underlying mechanisms by establishing sentinel sites with adequate temporal and spatial coverage [97]. National plans of action for insecticide resistance monitoring and management are recommended to encompass all related issues. By reviewing the insecticide resistance monitoring data from countries in the region, it was revealed that these recommendations are not fully implemented. To be more specific, in some provinces/states with ongoing malaria transmission, the susceptibility bioassay data are either old or even non existent, performed sub-standardly; in provinces/states with cleared up foci, monitoring insecticide susceptibility is grossly ignored whereas instead, it should be planned and performed, at least with a longer intervals, especially in areas with higher receptivity where malaria may be re-introduced. Another operational issue is the confirmation of resistance when it is considered ‘resistance to be confirmed’. Care must be taken not to treat this as an independent category of insecticide resistance status, but based on the WHO recommendations, additional tests must be performed to confirm the susceptibility status, an operation mostly forgotten. Determination of the intensity of resistance using 5× and 10× insecticide-treated papers is also emphasized to guide the programmes to choose the right insecticides [97]. Creating consortia of programmes, universities and research institutes for information and knowledge exchange between member states is encouraged to build the capacity of the region to adequately implement WHO recommendations regarding insecticide resistance monitoring and management.

DDT resistance in Iran and many other countries is primarily GST-related [43, 62, 84–86, 89]. On the other hand, GSTs may be involved secondarily in pyrethroid insecticide resistance in some insects [46, 100]. Therefore, wherever resistance to DDT is present, it means that GSTs levels may still be high, so care must be taken if pyrethroid insecticides are going to be used for vector control. Esterases overexpressed by upregulation, gene amplification as well as enhanced metabolic property [34, 90, 91, 101, 102] are involved mainly in organophosphorus and secondarily in carbamate and pyrethroid insecticides resistance. The involvement of esterases in insecticide resistance in *An. stephensi* from Iran, Afghanistan, India, and Pakistan is documented in several papers [34, 36, 47, 87, 89, 90, 102, 103]. The rather wide spectrum of impact of esterase-based insecticide resistance is of important operational implication as it defines the cross-resistance spectrum. Cytochrome p450 is also of high importance in conferring insecticide resistance primarily to pyrethroid insecticides and to a lesser extent to DDT, OPs and propoxur in many insect groups [44, 45]. Pyrethroid insecticide resistance in *An. stephensi* from Iran [78, 84–87], Afghanistan [27, 28] and India [34, 36] is in part due to cytochrome p450s. The involvement of p450s especially in pyrethroid insecticide resistance is of high operational implication and that is why WHO urges the malaria programmes to identify the involvement of cytochrome p450s in pyrethroid insecticide resistance using PBO-bioassay [97] to pave the way for possible PBO nets distribution. In such places IRS using pyrethroid insecticides plus PBO may also be feasible [78].

There are two types of *kdr* alleles in *Anopheles* mosquitoes, i.e., *kdr west* [33, 39, 103] and *kdr east* [26, 104]. However, there is a slight difference in those two alleles in terms of conferring resistance to DDT and pyrethroids [28, 105]. Therefore, not only it is important to identify the *kdr* alleles and its frequency in the mosquito populations under investigation, but their differential impact on phenotypic resistance should also be considered when planning for vector control interventions. The first report of *kdr* allele in *An. stephensi* was in the DUB-R laboratory strain [103]. Years later, several other studies detected *kdr west* and *east* mutations in *An. stephensi* in India [31, 38, 39, 48, 106] and Afghanistan [26–28]. The presence of *kdr* genotypes itself in low frequency may not jeopardize the effectiveness of pyrethroid insecticides especially in the form of LLINs [107–112]. However, if the trait is in high frequency and especially in combination with metabolic mechanisms, it may then impact the effectiveness of pyrethroid insecticides for malaria vector control as shown in a systematic review with meta-analysis [113]. This phenomenon again emphasizes the operational value of entomological surveillance especially monitoring insecticide resistance and its underlying mechanisms when planning malaria control.

Another important site insensitivity mechanism related to OPs and carbamates resistance is altered acetylcholine esterase (aAChE). A population is considered having phenotypic aAChE type mechanism if the frequency of the trait in the population is more than 60% [114]. Resistance to different OPs and carbamate insecticides in *An. stephensi* in Iran [87, 115] and Afghanistan [27, 28] can be traced back to aAChE. Therefore, the frequency of aAChE should be determined in order to guide the national malaria programme to choose the right insecticide for vector control.

In the face of insecticide resistance, attempts should be made to preserve the shelf life of public health pesticides two fold: first using insecticide resistance management strategies as well as through intersectoral collaboration, notably with agriculture sector as there are voluminous amounts of literature confirming the relationship between pesticide used in agriculture and emergence of insecticide resistance in disease vectors [95, 116–120]. The other fold being research and development for production of alternative insecticides or formulations for malaria vector control in the face of insecticide resistance especially to pyrethroids as they are only approved chemicals to be used in LLINs construction, several of these new molecules or formulations are now in the pipe line [73, 78, 121–127].

## Conclusions

*Anopheles stephensi* is an important, mostly urban, malaria vector in a wide range from Iraq to West China. In recent years, the species expanded its range to Sri Lanka and the Horn of Africa to Djibouti, Ethiopia and Sudan. The species is a leading malaria vector in terms of developing insecticide resistance. Since the development of DDT resistance in this vector in 1957 in Iran and in other countries in the region, voluminous amounts of literature have been produced on insecticide resistance in this species in EMR countries as well as in India. The species is now resistant to all major groups of insecticides with a different range of metabolic and site insensitivity mechanisms. As the countries of the range of distribution of this species, including the newly invaded ones, are in constant battle against malaria, continuous monitoring of insecticide resistance, its intensity and underlying mechanisms are essential to make evidence-based decisions when it comes to choosing vector control interventions. Also as insecticide resistance in *An. stephensi* is widespread, research and development on new formulations and molecules are essential to keep fighting malaria in countries of its range.

### Implication for practice

Monitoring susceptibility status of *An. stephensi* to insecticides is not routinely performed in sentinel sites and with adequate temporal and spatial coverage. This gap can harm malaria programmes in member states when it comes to decision-making for vector control in control, elimination and even post-elimination scenarios. The results of this review are important to be considered and implemented into practice by the national malaria programme, as insecticide resistance management is essential to choose appropriate insecticide for malaria vector control, while moving to the final steps of malaria elimination in Iran. The same is true for countries engaged in battle against *An. stephensi* for long time now or those countries and territories newly invaded by the species.

### Implication for research

Undertaking more research to elucidate the details of biochemical and molecular biology of the underlying mechanisms for insecticide resistance in *An. stephensi* in all countries of its range is highly encouraged. The results of research are crucial to keep the tools for vector control working to the final steps of malaria elimination in Iran and successful malaria vector control elsewhere. Research and development to produce lead molecules or new formulations is strongly recommended because of insecticide resistance, and because among other reasons, the number of available public health pesticides is limited.

## Data Availability

All data generated or analyzed during this study are included in this published article.

## Abbreviations

aAChE: altered acetylcholine esterase
AChE: acetylcholine esterase
DDT: dichlorodiphenyltrichloroethane
DMC: dichloromethyl benzhydrol
EMR: Eastern Mediterranean Region
GMEP: Global Malaria Eradication Program
GST: glutathione-S-transferases
IRS: Indoor Residual Spraying
*Kdr*: knockdown resistance
LLIN: long-lasting insecticidal nets
OPs: organophosphates
PBO: piperonyl butoxide
WHO: World Health Organization
